# The Effects of Physical Training on Quality of Life, Aerobic Capacity, and Cardiac Function in Older Patients With Heart Failure: A Meta-Analysis

**DOI:** 10.3389/fphys.2018.01564

**Published:** 2018-11-12

**Authors:** Maamer Slimani, Rodrigo Ramirez-Campillo, Armin Paravlic, Lawrence D. Hayes, Nicola Luigi Bragazzi, Maha Sellami

**Affiliations:** ^1^Department of Health Sciences (DISSAL), School of Public Health, Genoa University, Genoa, Italy; ^2^Laboratory of Human Performance, Department of Physical Activity Sciences, Research Nucleus in Health, Physical Activity and Sport, Universidad de Los Lagos, Osorno, Chile; ^3^Science and Research Centre, Institute for Kinesiology Research, Garibaldijeva, Koper, Slovenia; ^4^Active Ageing Research Group, University of Cumbria, Lancaster, United Kingdom; ^5^Sport Science Program, College of Arts and Sciences (QU-CAS), University of Qatar, Doha, Qatar

**Keywords:** exercise training, resistance training, physical function, health status, cardiac rehabilitation

## Abstract

**Aim:** The purposes of this meta-analysis were to quantify the effectiveness of physical training on quality of life (QoL), aerobic capacity, and cardiac functioning in older patients with heart failure (HF) and evaluate dose–response relationships of training variables (frequency, volume, and duration).

**Methods:** Scholarly databases (e.g., PubMed/MEDLINE, Google Scholar, and Scopus) were searched, identifying randomized controlled trials that investigated the effectiveness of different training modes on QoL (assessed by the Minnesota Living with Heart Failure Questionnaire), aerobic capacity (assessed by the 6 min walk test) and cardiac function (assessed by left ventricular ejection fraction).

**Results:** Twenty five studies were included with a total of 2,409 patients. Results showed that exercise training improved total QoL (small ES = −0.69; 95% CI −1.00 to 0.38; *p* < 0.001), aerobic capacity (small ES = 0.47; 95% CI 0.15–0.71; *p* = 0.002) and cardiac function (moderate ES = 0.91; 95% CI 0.37–1.45; *p* = 0.001). In addition, univariate analyses revealed the moderating variable ‘training mode' significantly influenced aerobic capacity (*Q* = 9.97; *p* = 0.007), whereby, resistance training had the greatest effect (ES = 1.71; 95% CI 1.03–2.39; *p* < 0.001), followed by aerobic training (ES = 0.51; 95% CI 0.30–0.72; *p* < 0.001), and combined training (ES = 0.15; 95% CI −0.24 to 0.53; *p* = 0.45). Meta-regression analysis showed that only the duration of an intervention predicted the effect of physical training on QoL (coefficient = −0.027; *p* = 0.006), with shorter training durations (12 weeks) showing larger improvements.

**Conclusion:** The present meta-analysis showed that physical training has positive effects on QoL, aerobic capacity, and cardiac function in older patients with HF. Practitioners should consider both training volume and mode when designing physical training programs in order to improve QoL and aerobic capacity in older patients with HF.

## Introduction

Heart failure (HF), a complex clinical syndrome characterized by reduced ability of the heart to pump and/or fill with blood, represents a major public health problem, with a computed prevalence of over 5.8 million in the USA, and over 23–26 million worldwide (Roger, 2013). Being a global pandemic, prevalence is still increasing and is expected to reach 8 million people in the USA by 2030, whereas up to 15 million people are living with HF in Europe (Maggioni, 2015). In Italy, the burden is especially prevalent among elderly people and in Regions such as Liguria, which is a Region with Europe's oldest residents (Marangoni et al., 2012).

As such, HF imposes high societal costs and impacts on patient quality of life (QoL) (Heo et al., 2009). QoL relates to a multitude of aspects such as physical fitness, social environment, education, employment, economic and finance conditions, and other markers such as religion and beliefs, and environment (Katschnig, 1997; Coelho et al., 2005). Among these elements, physical and mental health play an important role in management and characterization of QoL of a person (Rodríguez-Fernández et al., 2017), and especially a patient with cardiac disease (Pedersen et al., 2007). In fact, it has been shown that low pre-hospitalization health-related QoL and poor fitness are associated with lower post-hospitalization general health, increased remission rates, and mortality risk (Mendes de Leon et al., 1998; Hawkes et al., 2013).

Prevention of cardiac disease based on physical activity plays a major role in public health, and physical activity should be considered a priority in prevention of chronic-degenerative disorders (Romano-Spica et al., 2015). Regular physical activity mitigates the risk of HF hospitalization and mortality (Hegde et al., 2017). There seems to be a strong, dose-dependent association between physical activity/fitness and the risk of HF. As such, exercise can be conceived as a non-pharmacological treatment to counter the pathophysiological mechanisms leading to HF (Pandey et al., 2015).

Physical activity generally improves QoL, aerobic fitness, and cardiac function in HF patients (Beniaminovitz et al., 2002; Harris et al., 2003; Gary et al., 2004; Koukouvou et al., 2004; Davidson et al., 2010; Hassanpour Dehkordi and Khaledi Far, 2015). However, this is not always the case, as Keteyian et al. (1999) showed no significant change in QoL after 6 months' aerobic training in male HF patients.

Different modes of training (namely aerobic, resistance, and combined training) result in divergent physiological adaptations, and the most beneficial mode for HF patients remains unclear. Delagardelle et al. (2002) and Mandic et al. (2009) reported that combined aerobic and resistance training was superior to aerobic training exclusively for improving cardiorespiratory fitness (i.e., peak oxygen uptake [VO_2_peak]). In contrast, Haykowsky et al. (2005) reported no significant difference between combined aerobic and resistance training and aerobic training only in cardiorespiratory fitness (i.e., VO_2_peak), and therefore, ambiguity remains as to which modality of training is optimal for patients with HF. Moreover, most investigations report no difference between short- and long-term training for QoL and cardiorespiratory fitness (i.e., 6 min walk test [6-MWT]) improvement (Dracup et al., 2007; Jolly et al., 2009; Davidson et al., 2010).

Previous meta-analyses have shown exercise training improves cardiorespiratory fitness, ejection fraction and QoL in cardiac patients (Piepoli et al., 2004; Smart and Marwick, 2004; Haykowsky et al., 2007; Giuliano et al., 2017; Ostman et al., 2017). More specifically, Haykowsky et al. (2007) reported that aerobic training improved ejection fraction in patients with HF to a greater extent than combined aerobic and strength training. Furthermore, Ostman et al. (2017) reported that high-intensity physical training reduced total QoL score (i.e., improved QoL). However, despite the existing meta-analyses in the field, whether there is a most effective training mode and/or dose-response relationship remains unknown. The effectiveness of physical training on QoL, aerobic capacity, and left ventricular ejection fraction in older HF patients in terms of training variables (frequency, volume, and duration) is still unknown. This knowledge would allow practitioners to (i) maximize training-related health benefits and athletic performance and (ii) adequately design strength and conditioning programs for cardiac rehabilitation.

Therefore, the present meta-analysis was designed in order to fill the aforementioned void in knowledge. In particular, the aim of this meta-analysis was to establish the effects of physical training on QoL, aerobic capacity, and left ventricular ejection fraction in older HF patients. A secondary aim was to quantify dose-response relationships according to training modalities and program variables.

## Materials and methods

### Search strategy

**Figure 1:**
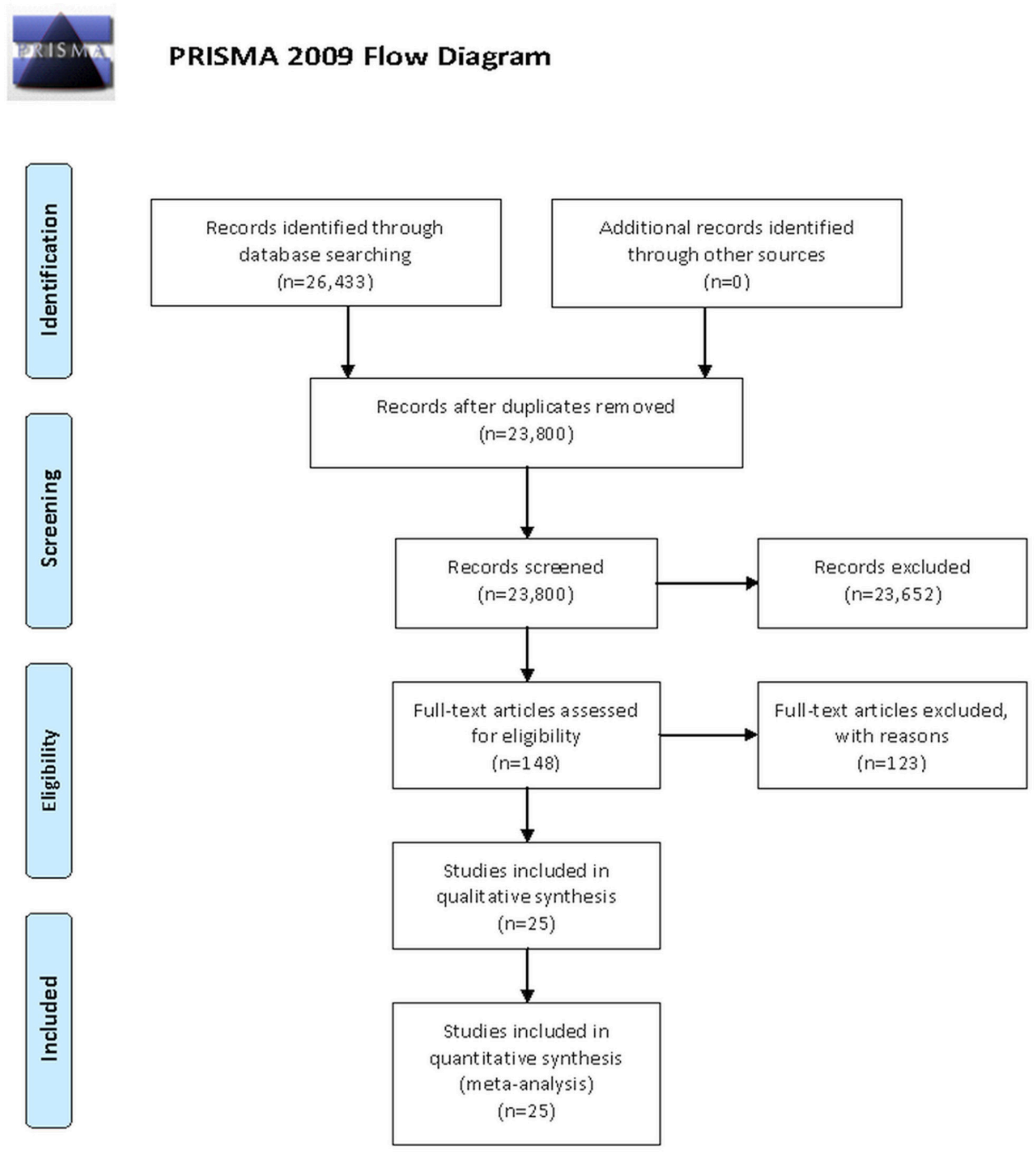
Flowchart of the process of search strategy adopted in the present meta-analysis.

This meta-analysis was conducted according to the Preferred Reporting Items for Systematic Reviews and Meta-Analysis (PRISMA) guidelines (Figure 1, Moher et al., 2009). A systematic literature search was conducted for randomized controlled trials (RCTs) studying the effects of physical training on QoL in older patients with HF. Studies were obtained through systematic manual and electronic searches (up to May 1st, 2018) in electronic databases (i.e., Google Scholar, MEDLINE/PubMed, and Scopus). Electronic databases were searched using the following search syntax with keywords and/or MeSH terms: [(“aerobic training” OR “resistance training” OR “power training” OR “plyometric training” OR “exercise”) AND (“elderly” OR “older”) AND “heart failure” AND (“quality of life” OR “Minnesota Living with Heart Failure Questionnaire” OR “walking test” OR “left ventricular ejection fraction”)]. Moreover, we performed manual searches of relevant journals and reference lists obtained from published articles. The present meta-analysis included studies published in journals that reported original research data from older patients with HF.

### Inclusion and exclusion criteria

Studies were included in this meta-analysis if they met all the following Population/Intervention/Comparison/Outcome(s) (PICOS) criteria:

(1) Population: studies recruiting older patients with HF as participants from different countries (i.e., Belgium, Brazil, Canada, Greece, Iran, Italy, Netherlands, Sweden, Taiwan, United States, United Kingdom); Older patient groups includes the younger old (65–74 years), the old (75–84 years), and the older old or oldest old (>85 years) (Little et al., 2012). However, we allowed 50 years of age as a minimum reference range of age for studies on African population (World Health Organization, 2016), and from 65 years for populations from developed countries (Kowal and Dowd, 2001).
(2) Intervention or exposure:
  a) Studies examining the effects of physical training on QoL, aerobic capacity, and cardiac function in older patients with HF;
  b) Studies describing their training variables (e.g., volume, frequency, and duration).

(1) Comparison: Studies involving a control group against which an intervention was compared.
(2) Outcome(s): QoL, aerobic capacity, or cardiac function assessed using the Minnesota Living with Heart Failure Questionnaire (MLWHFQ), the 6-MWT (i.e., total distance covered), and left ventricular ejection fraction, respectively. In addition, we examined how moderating variables like training duration (weeks), training frequency (sessions/week), and type of training, influenced physical training related QoL, aerobic capacity and cardiac function enhancements.
(3) Study design: original research in the form of RCTs.
  Studies were excluded if:
  (i) They were reviews, opinion papers and commentaries, interviews, letters to the editor, editorials, posters, conference papers, abstracts, book chapters, or books. However, published review articles were examined to avoid missing relevant articles;
  (ii) They did not include sufficient data to calculate standardized mean differences.

### Coding of studies

Two authors independently extracted data using a structured form. Because of the high number of variables that may affect training effectiveness, independent variables were grouped into the following areas as reported in the included studies: (i) training modes (aerobic vs. resistance vs. combined aerobic and resistance) and (ii) training variables (training duration in weeks [4–8 weeks vs. 12 weeks vs. 16 weeks vs. 5–6 months vs. 1 year], weekly training frequency [1–2 vs. 3 vs. 4 vs. 5 or more sessions per week], and session duration (20–30 min vs. 31–45 min vs. 46–65 or more min).

### Data extraction

The main study characteristics (i.e., intervention program, training variables, relevant outcomes) were extracted into a Microsoft Excel/spreadsheet.

### Statistical analyses

Data were extracted from the included studies using a standardized documentation form. Effect size (ES) and 95% confidence intervals (95% CI) were calculated for the identified studies. Meta-analyses were computed using the program Comprehensive Meta-Analysis, version 2 (Borenstein et al., 2005). Statistical heterogeneity was assessed using *Q* and *I*^2^. The *I*^2^ measure of inconsistency was used to examine between-study variability. Values of 25, 50, and 75% represent low, moderate, and high statistical heterogeneity, respectively (Higgins et al., 2003). Due to study heterogeneity, we applied a random-effects model for all comparisons. Potential publication bias was visually inspected with a funnel plot, looking at asymmetry of the graph. In addition, meta-regression analyses (method of moments) were applied to compute possible predictors that may have influenced training-related effects (e.g., training duration, weekly training frequency, and session duration). Effect sizes (ES) were classified as trivial (< 0.35), small (0.35–0.80), moderate (0.80–1.50), or large (>1.5) (Rhea et al., 2003) and significance level was set *a priori* at *p* < 0.05.

## Results

### Search results

The applied search strategy yielded a preliminary number of 26,433 studies. After removing duplicates, 23,800 unique studies were screened. Screening of titles and abstracts resulted in 23,652 papers being discarded. This was due to the nature itself of the search strategy, which was designed to be the broadest possible in order to capture all relevant studies and was performed utilizing different scholarly databases, including the gray literature. Full texts of 148 articles were retrieved and assessed using the predetermined inclusion and exclusion criteria. After a careful review of full texts, 123 articles were excluded and the remaining 25 articles were included in this meta-analysis. A flow chart of the systematic search process is illustrated in Figure 1. Details of all included studies are depicted in Tables 1A–C.

**Table 1a:** Effect of physical training on quality of life.

**Study**	**Sex**	**Training mode**	**Training period (weeks)**	**Session duration (min)**	**Number of sessions per week**	**Exercise group**						**Control group**					
						**Before**			**After**			**Before**			**After**		
						***N***	**Mean**	**SD**	***N***	**Mean**	**SD**	***N***	**Mean**	**SD**	***N***	**Mean**	**SD**
Beniaminovitz et al., 2002	Both	Combined	4	30	3	17	50	3.8	17	37	3	8	41	4.6	8	33	3.5
Harris et al., 2003	Both	Resistance	6	30	5	24	36.3	4.21	24	31	3.66	22	28.7	4.7	22	25.5	4.61
Chien et al., 2011	Both	Combined	8	30	3	24	11	11	22	7	9	27	16	16	22	13	13
Davidson et al., 2010	Both	Aerobic	16	60	1	53	44.11	23.7	50	27.9	12.46	52	52.37	26.38	44	36.89	16.22
Kitzman et al., 2013	Both	Aerobic	16	60	3	24	36	19	24	26	19	30	28	23	30	25	22
Kitzman et al., 2010	Both	Aerobic	16	60	3	26	32	20	26	25	24	27	25	22	27	27	19
Edelmann et al., 2011	Both	Combined	16	30	2–3	44	14	14	44	9	8	20	13	0	20	11	9
Brubaker et al., 2009	Both	Aerobic	16	60	3	30	39.9	4.2	30	25.3	4.2	29	44.1	4.9	29	37.9	4.2
Chrysohoou et al., 2014	Both	Aerobic	12	45	3	33	21	7	33	7	9	39	19	12	39	21	13
Dracup et al., 2007	Both	Combined	12	45	4	86	46.7	23.8	86	37.5	23.9	87	49.2	22.4	87	46.7	26.5
McKelvie et al., 2002	Both	Combined	12	30	2–3	91	32.5	2.5	70	28.6	1.9	90	28.6	2.1	73	27.4	1.5
Gary and Lee, 2007	Female	Aerobic	12	25		13	38	26	13	20	16	10	24	16	10	25	18
Fu et al., 2016	Both	Aerobic	12	32.5	3	30	41	2.1	30	22.5	2.3	30	42	2.6	30	40.1	2.8
Fu et al., 2016	Both	Aerobic	12	32.5	3	30	42.1	2.5	30	24.1	3.1	30	41.3	2.3	30	41.8	2.8
Mandic et al., 2009	Both	Aerobic	12	30	3	14	45.9	16.8	14	41.4	23.2	13	40.2	22.5	13	37.8	24.7
Mandic et al., 2009	Both	Combined	12		3	15	40	19.8	15	32.6	20.2	13	40.2	22.5	13	37.8	24.7
Patwala et al., 2009	Both	Aerobic	12	30	3	25	34.6	22.3	25	26.2	20.5	25	29	16.3	25	29.5	17.8
Maria Sarullo et al., 2006	Both	Aerobic	12	30	3	30	31.2	2.2	30	35.5	1.9	30	30.6	2.5	30	31.2	2.6
Servantes et al., 2012	Both	Aerobic	12	57.5	3–4	17	40.4	17.9	17	20.7	16.3	11	46.5	18.5	11	51	16.8
Servantes et al., 2012	Both	Combined	12		3–4	17	45.1	20.8	17	25.1	16.5	11	46.5	18.5	11	51	16.8
van den Berg-Emons et al., 2004	Both	Aerobic	12	60	2	18	24.1	19.7	18	18.1	18.5	16	27.5	13.9	16	26.5	12.7
Davidson et al., 2010	Both	Aerobic	54	60	1	53	44.11	23.7	50	52.9	15.68	52	52.37	26.38	42	56.43	18.28
Jolly et al., 2009	Both	Combined	54	25	5	84	33.35	21.3	84	37.61	20.97	85	31.82	22.5	85	34.91	24.8
McKelvie et al., 2002	Both	Combined	54	30	2–3	91	32.5	2.5	57	29.1	2.4	90	28.6	2.1	67	25.3	1.7
Pihl et al., 2011	Both	Resistance	54	45	3–4	28	22.4	15.5	28	25.2	10	31	24	17.1	31	25.3	11
Conraads et al., 2007	Both	Aerobic	20	60	3	8	51	9	8	30	6	9	36	8	9	24	7
Hassanpour Dehkordi and Khaledi Far, 2015	Both	Aerobic	24	40	3	30	65.12	15.93	30	70.28	19.33	31	64.85	17	31	57.92	18.5
Dracup et al., 2007	Both	Combined	24	45	4	86	46.7	23.8	86	35.7	23.7	87	49.2	22.4	87	43.2	27.3
Evangelista et al., 2017	Both	Combined	24	27.5	5	19	48	18.1	19	39.1	20.4	27	46.9	21	27	49.4	25
Evangelista et al., 2017	Both	Combined	24	27.5	5	16	43.9	22.9	16	36.7	21.7	27	46.9	21	27	49.4	25
Evangelista et al., 2017	Both	Combined	24	27.5	5	9	45.5	22.9	9	47.7	20.9	27	46.9	21	27	49.4	25
Jolly et al., 2009	Both	Combined	24	25	5	84	33.35	21.3	84	36.26	24.08	85	31.82	22.5	85	34.49	23.98
Keteyian et al., 1999	Male	Aerobic	24	33	3	21	38	28	21	31	22	22	28	18	22	26	23
Koukouvou et al., 2004	Male	Aerobic	24	60	3–4	16	45.5	17.1	16	34.1	13	10	45.1	9.9	10	45.2	9

**Table 1b:** Effect of physical training on aerobic capacity (i.e., 6 min walk test).

**Study**	**Sex**	**Training mode**	**Training period (weeks)**	**Session duration (min)**	**Number of sessions per week**	**Exercise group**						**Control group**					
						**Before**			**After**			**Before**			**After**		
						***N***	**Mean**	**SD**	***N***	**Mean**	**SD**	***N***	**Mean**	**SD**	***N***	**Mean**	**SD**
Harris et al., 2003	Both	Resistance	6	30	5	24	459	24	24	540	23	22	491	26	22	531	25
Chien et al., 2011	Both	Combined	8	30	3	24	424	145	22	433	145	27	432	81	22	429	93
Davidson et al., 2010	Both	Aerobic	16	60	1	53	279.45	110.97	50	361.2	132.34	52	251.06	112.57	42	274.98	106.6
Kitzman et al., 2013	Both	Aerobic	16	60	3	24	447	107	24	486	89	30	438	79	30	448	70
Edelmann et al., 2011	Both	Combined	16	30	2–3	44	545	86	44	569	88	20	551	86	20	568	80
Chrysohoou et al., 2014	Both	Aerobic	12	45	3	33	422	77	33	476	82	39	406	64	39	423	65
McKelvie et al., 2002	Both	Combined	12	30	2–3	91	434	7	70	456	12	90	421	8	73	436	13
Gary et al., 2004	Female	Aerobic	12	25		13	914	362	13	923	346	10	817	422	10	811	391
van den Berg-Emons et al., 2004	Both	Aerobic	12	60	2	18	455	71	18	501	96	16	435	77	16	448	84
Davidson et al., 2010	Both	Aerobic	54	60	1	53	279.45	110.97	47	386.55	129.97	52	251.06	112.57	33	247.27	122.96
McKelvie et al., 2002	Both	Combined	54	30	2–3	91	434	7	70	451	15	90	421	8	67	441	17

**Table 1c:** Effect of physical training on cardiac function (Left ventricular ejection fraction [%]).

**Study**	**Sex**	**Training mode**	**Training period (weeks)**	**Session duration (min)**	**Number of sessions per week**	**Exercise group**						**Control group**					
						**Before**			**After**			**Before**			**After**		
						***N***	**Mean**	**SD**	***N***	**Mean**	**SD**	***N***	**Mean**	**SD**	***N***	**Mean**	**SD**
Kitzman et al., 2013	Both	Aerobic	16	60	3	24	58	6	24	58	6	30	56	5	30	56	5
Kitzman et al., 2010	Both	Aerobic	16	60	3	26	61	5	26	57	8	27	60	10	27	55	8
Edelmann et al., 2011	Both	Combined	16	30	2–3	44	67	7	44	66	6	20	66	7	20	67	8
Brubaker et al., 2009	Both	Aerobic	16	60	3	30	31.1	2.2	30	29.4	1.8	29	32.8	2.4	29	29.1	2.3
McKelvie et al., 2002	Both	Combined	12	30	2–3	91	28.2	0.8	80	28.5	1.5	90	27.7	0.9	81	29.3	1.6
Fu et al., 2016	Both	Aerobic	12	32.5	3	30	57.6	1.9	30	57.8	1.7	30	56.5	2.2	30	54.4	3.3
Fu et al., 2016	Both	Aerobic	12	32.5	3	30	28.1	1.1	30	46.2	2.5	30	28.1	1.4	30	28	1.2
Mandic et al., 2009	Both	Aerobic	12	30	3	7	30.9	12.2	7	33.2	12.6	8	28.9	11.9	8	28.4	9.2
Mandic et al., 2009	Both	Combined	12		3	15	32.7	14.7	15	35.4	10.1	13	28.9	11.9	8	28.4	9.2
Patwala et al., 2009	Both	Aerobic	12	30	3	25	32.8	6.2	25	37.3	5.4	25	32.6	7	25	35	7.2
Maria Sarullo et al., 2006	Both	Aerobic	12	30	3	30	29.2	5	30	30.1	4	30	28.9	4	30	27.3	4
Conraads et al., 2007	Both	Aerobic	20	60	3	8	27	5	8	36	5	9	28	5	9	34	6
Hassanpour Dehkordi and Khaledi Far, 2015	Both	Aerobic	24	40	3	30	32	4	30	37	5	31	33	5	31	31	5

### Effects of physical training on quality of life in patients with heart failure

**Figure 2:**
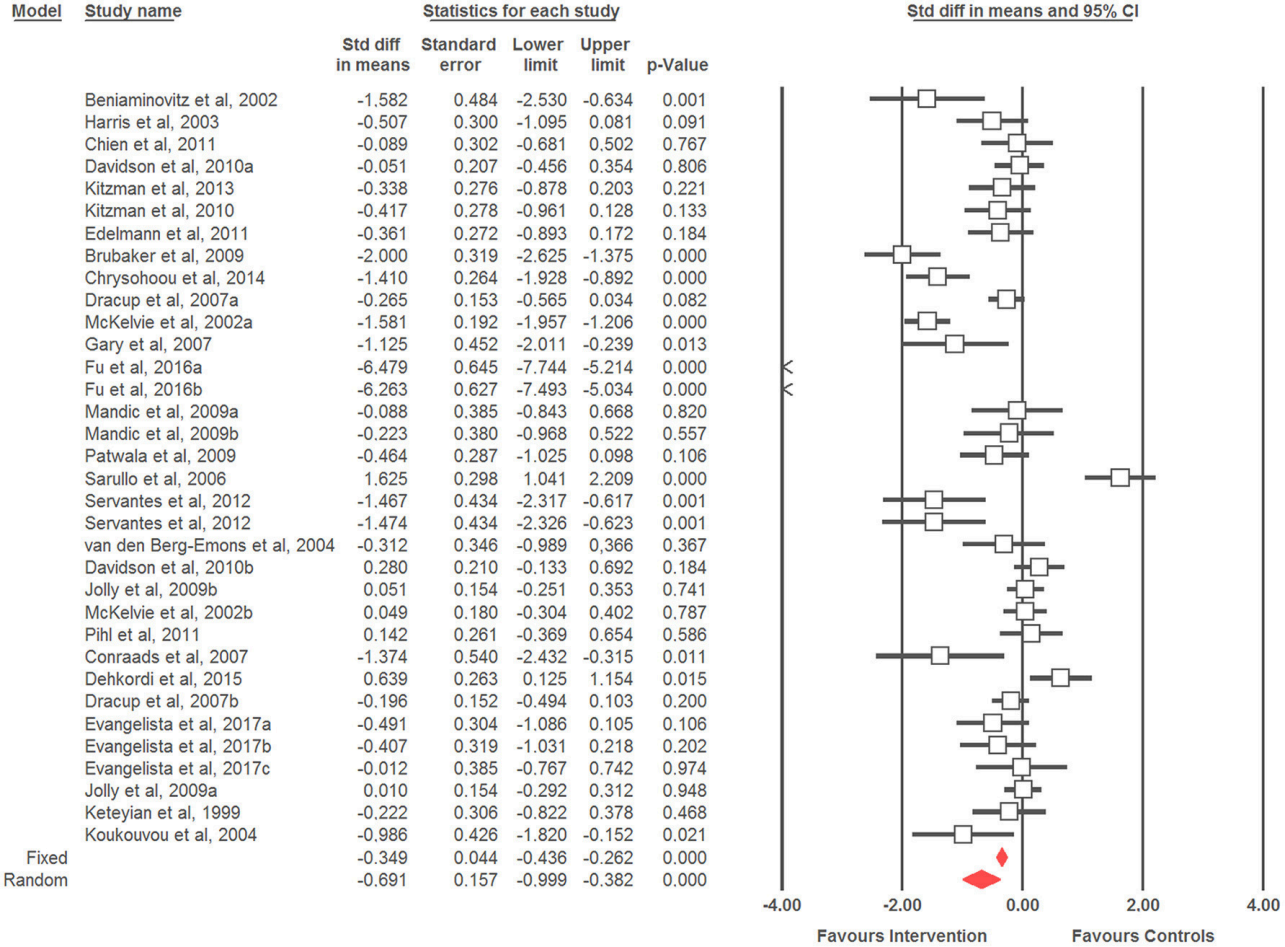
Forest plot of physical training effect on quality of life of older patients with HF.

Twenty five studies totalizing 34 ES were identified and a small effect of physical training on QoL was observed (ES = −0.69; 95% CI = −1.00 to −0.38; *p* < 0.001) (Figure 2). High heterogeneity was observed (*I*^2^ = 91.45%; *p* < 0.001). Therefore, sub-group analysis was conducted, observing a moderate QoL improvement in females (ES = −1.13; 95% CI = −2.01 to −0.24; *p* = 0.013), small QoL improvement in males (ES = −0.55; 95% CI = −1.29 to 0.19; *p* = 0.148), and small QoL improvement in males and females combined (ES = −0.69; 95% CI −1.02 to −0.36; *p* < 0.001), without significant difference between them (*Q* = 1.05; *p* = 0.592). When the effects of different intervention types were analyzed, QoL improvements were observed following aerobic training (moderate ES = −1.04; 95% CI = −1.67 to −0.41; *p* = 0.001) and combined aerobic and resistance training (small ES = −0.42; 95% CI −0.71 to −0.13; *p* = 0.005), with trivial effects after resistance training (ES = −0.17; 95% CI = −0.80 to 0.47; *p* = 0.610). However, no significant difference was observed between training modes (*Q* = 4.20; *p* = 0.123) (Table 2).

**Table 2:** Effects of physical training on quality of life, aerobic capacity, and cardiac function in older patients with HF patients considering different moderating variables.

**Independent variables**	**SMD**	**SE**	**95% CI**	***p***	***I^2^* (%)**	***df***	***Q value and (p) between groups***
**QUALITY OF LIFE**							
**Gender**							1.05 (0.592)
Males	−0.55	0.38	−1.29 to 0.19	0.148	52.92	1
Females	−1.33	0.45	−2.01 to−0.24	**0.013**	0.0	0
Combined	−0.69	0.17	−1.02 to −0.36	<**0.001**	92.12^**^	30
**Training mode**
aerobic	−1.04	0.32	−1.67 to−0.41	**0.001**	94.44^**^	17	4.20 (0.123)
Resistance	−0.17	0.32	−0.80 to 0.47	0.610	62.53	1
Combined	−0.42	0.15	−0.71 to −0.13	**0.005**	82.48^**^	13
**AEROBIC CAPACITY**							
**Gender**
Females	0.04	0.42	−0.78 to 0.87	0.922	0.0	0	0.88 (0.349)
Combined	0.46	0.15	0.17 to 0.75	**0.002**	73.34^**^	9
**Training mode**
Aerobic	0.51	0.11	0.30 to 0.72	<**0.001**	0.00	5	15.54 (< **0.001**)
Resistance	1.71	0.34	1.03 to 2.39	<**0.001**	0.00	0
Combined	0.15	0.20	−0.24 to 0.53	0.458	69.12^*^	3
**CARDIAC FUNCTION**							
**Training mode**
Aerobic	1.17	0.37	0.45 to 1.89	**0.001**	92.16^**^	9	2.49 (0.115)
Combined	0.31	0.41	−0.49 to 1.10	0.450	84.71^**^	2

*SMD, standardized mean difference; SE, standard error; CI, confidence interval; p, significance value; I^2^, heterogeneity index; df, degrees of freedom. Bold values indicate statistically significant values*.

* *p < 0.05*;

** *p < 0.01*.

### Effects of physical training on aerobic capacity and cardiac function in older patients with heart failure

**Figure 3:**
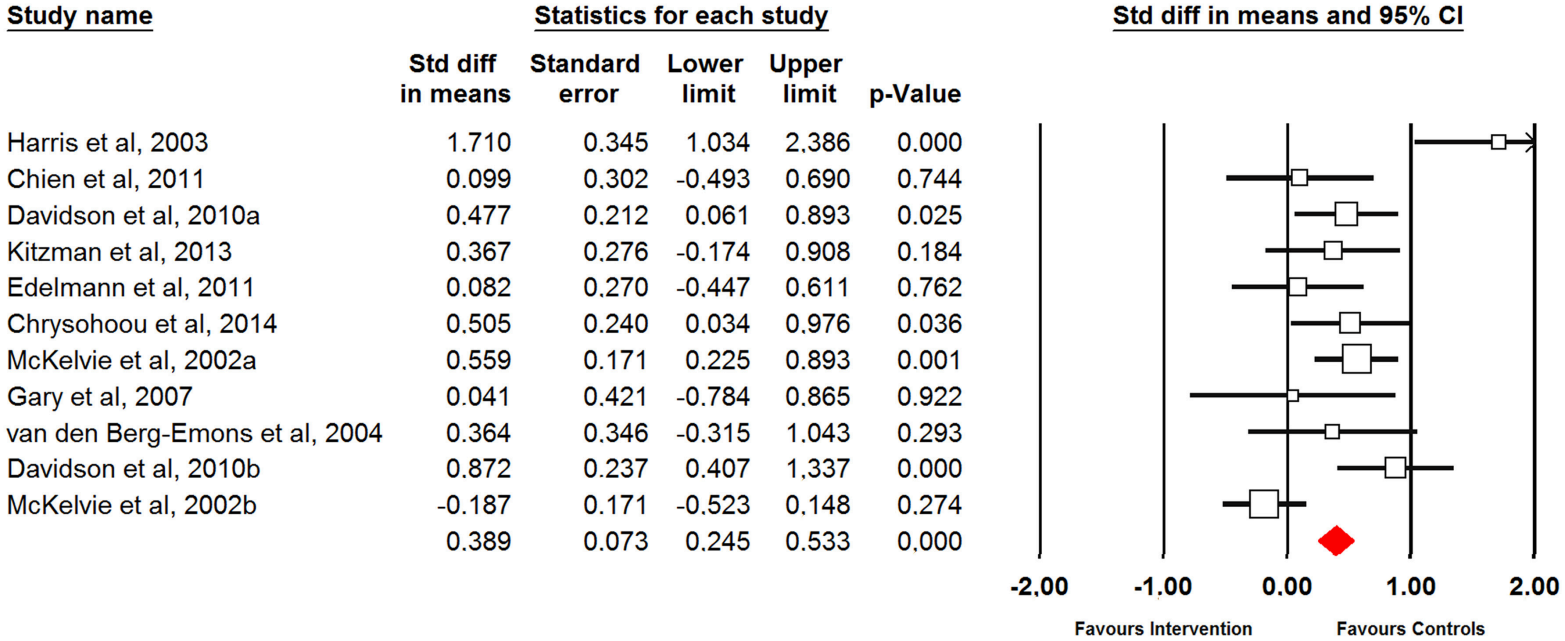
Forest plot of physical training effect on aerobic capacity of older patients with HF.

Eleven studies examined the effects of physical training on aerobic capacity (i.e., total distance covered in the 6-MWT). The grouped effect (i.e., all type of physical training combined) revealed improvements following intervention (small ES = 0.43; 95% CI = 0.15 to 0.71; *p* = 0.002) (Figure 3). Small improvements were observed in males and females combined (ES = 0.46; 95% CI = 0.17–0.75; *p* = 0.002), with trivial effect on females (ES = 0.04; 95% CI = −0.78 to 0.87; *p* = 0.922). Due to insufficient data, the effect on males was not calculated. When different types of physical training were analyzed, large improvements were observed following resistance training (ES = 1.71; 95% CI = 1.03 to 2.39; *p* < 0.001), small improvements were observed following aerobic training (ES = 0.51; 95% CI = 0.30 to 0.72; *p* < 0.001), and trivial improvements were observed following combined aerobic and resistance training (ES = 0.15; 95% CI = −0.24 to 0.53; *p* = 0.458). Additionally, significant difference was observed between different training modes (*Q* = 15.54; *p* < 0.001) (Table 2).

**Figure 4:**
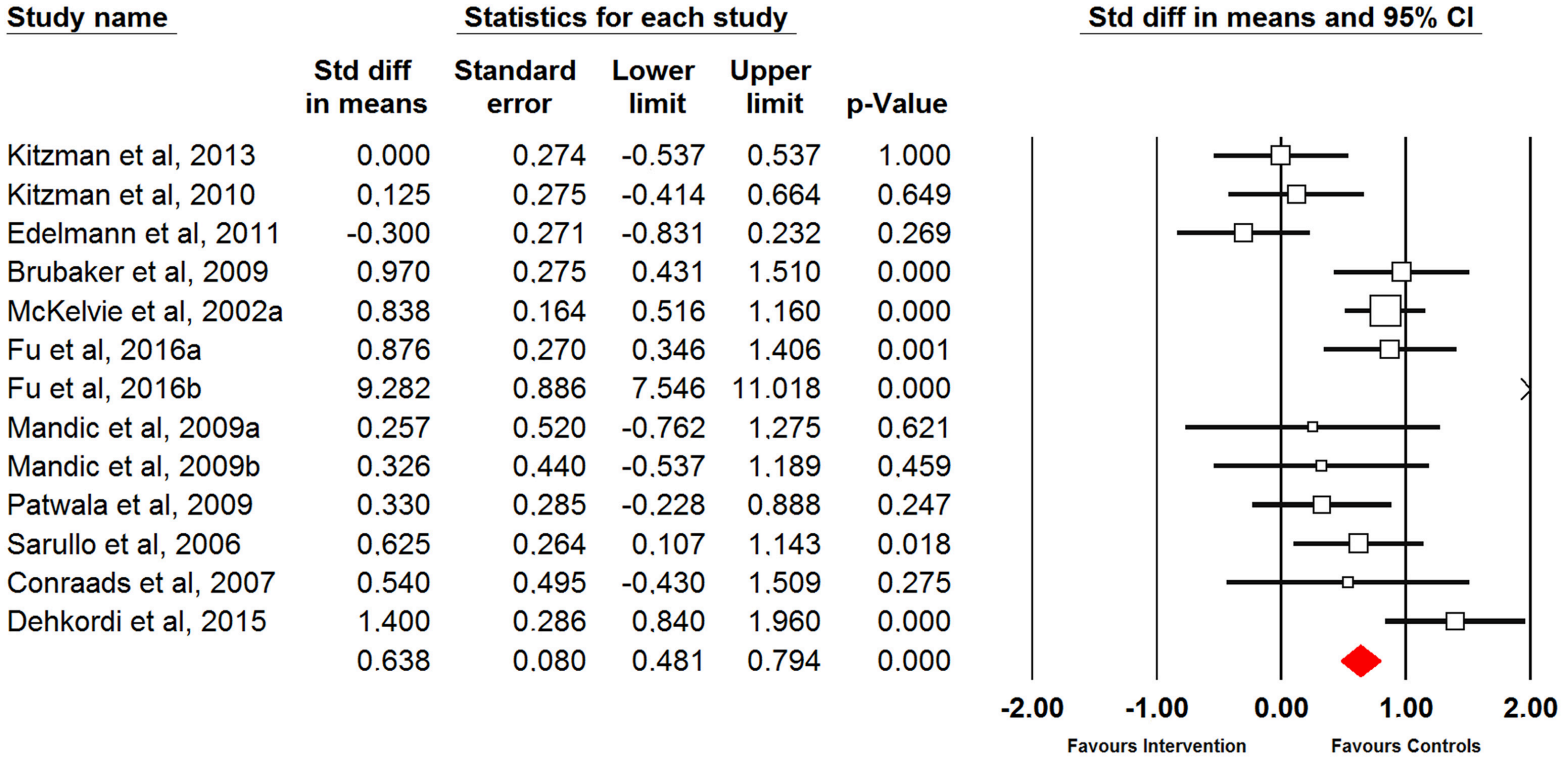
Forest plot of physical training effect on cardiac function of heart failure patients.

Cardiac function (i.e., left ventricular ejection fraction) showed moderate improvements after physical training (moderate ES = 0.91; 95% CI = 0.37 to 1.45; *p* = 0.001) (Figure 4). When different interventions were analyzed, there were improvements following aerobic training (moderate ES = 1.17; 95% CI = 0.45 to 1.89; p = 0.001) and combined aerobic and resistance training (small ES = 0.30; 95% CI = −0.49 to 1.10; *p* = 0.450), without significant difference between training modes (*Q* = 2.49; *p* < 0.115) (Table 2).

### Dose-response relationship of physical training on quality of life, aerobic capacity, and cardiac function in older patients with heart failure

#### Findings from meta-regression analysis

**Figure 5:**
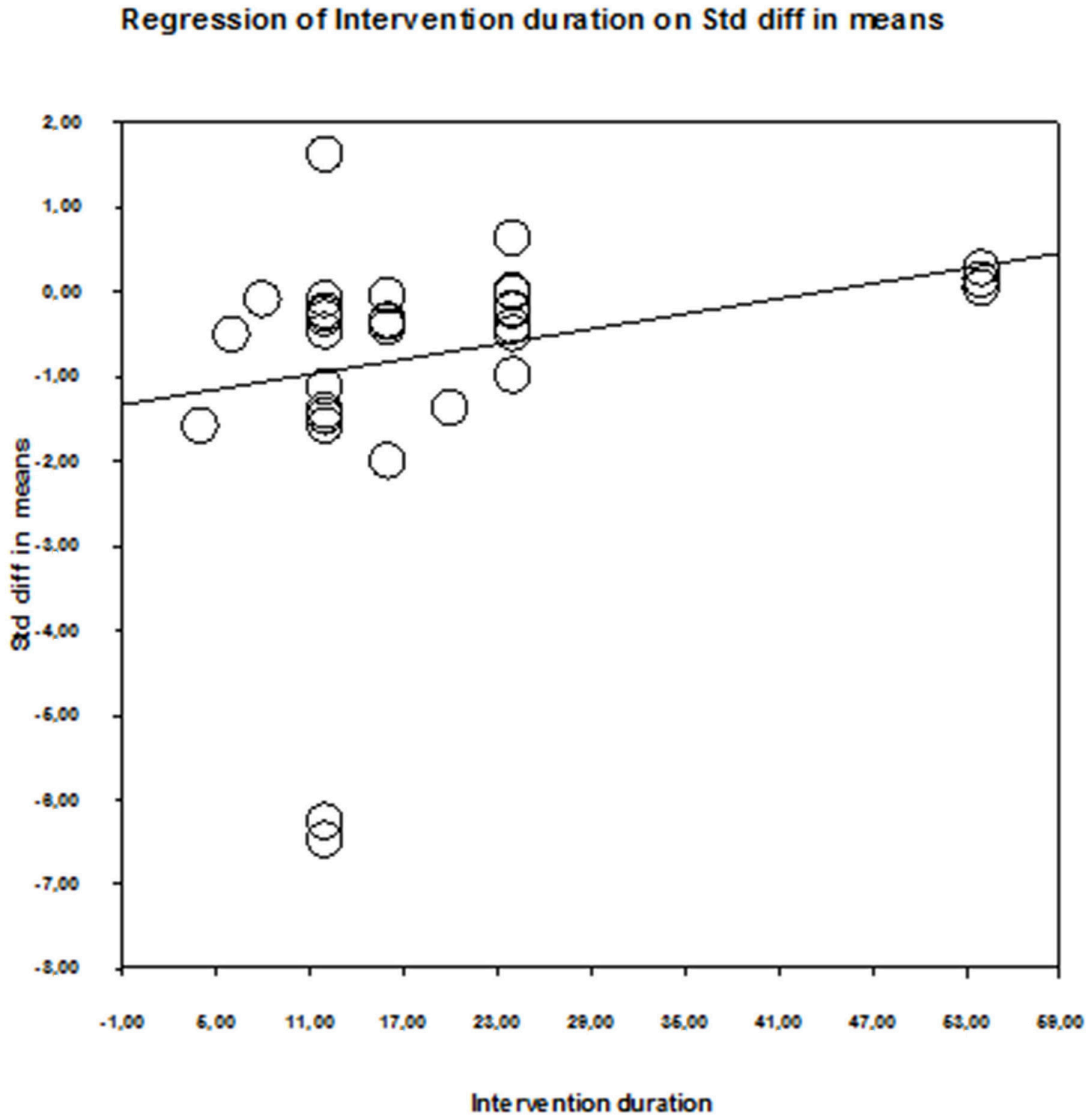
Scatter plot of regression analysis showing influence of intervention duration on the quality of life of older patients with HF.

Table 3 shows the results of the meta-regression for the three training variables: duration of intervention, duration of single session, and weekly frequency. Only the duration of the intervention predicted QoL changes after physical training (*p* = 0.006) (Figure 5). The predictive influence of the remaining training variables was *p* = 0.665–0.996. Therefore, none of the examined training variables predicted aerobic capacity and cardiac function adaptation (*p* = 0.280–0.522) (Table 3).

**Table 3:** Meta regression for training variables of different subscales to predict physical training effect on quality of life, aerobic capacity and cardiac function in in older patients with HF.

	**Beta coefficient**	**Standard error**	**95% lower CI**	**95% upper CI**	***Z*-value**	***P*-value**
**QUALITY OF LIFE**						
Duration of intervention	0.030	0.011	0.008	0.051	2.739	**0.006**
Duration of single session	0.000	0.012	−0.024	0.024	−0.004	0.996
Weekly frequency	0.068	0.158	−0.241	0.378	−0.433	0.665
**AEROBIC CAPACITY**						
Duration of intervention	−0.006	0.008	−0.022	0.010	−0.699	0.484
Duration of single session	0.006	0.010	−0.013	0.026	0.641	0.522
Weekly frequency	0.161	0.149	−0.131	0.452	1.079	0.280
**CARDIAC FUNCTION**						
Duration of intervention	−0.053	0.079	−0.209	0.102	−0.675	0.500
Duration of single session	−0.069	0.083	−0.232	0.094	−0.825	0.409
Weekly frequency	1.579	1.607	−1.570	4.727	0.983	0.326

*Bold values indicate statistically significant values*.

#### Findings from the univariate analysis

Sub-analysis (Table 4) revealed that 12 weeks' training induced the greatest improvements in QoL (ES = −1.41; 95% CI = −2.20 to −0.61; *p* = 0.001). Regarding the frequency of training, 3–4 sessions per week induced the greatest improvements in QoL (ES = −0.98; 95% CI = −1.49 to −0.48; *p* < 0.001). Regarding single session duration, 31–45 min of training per session induced the greatest improvements in QoL (ES = −1.57; 95% CI = −2.53 to −0.60; *p* = 0.001).

**Table 4:** Effects of physical training on quality of life considering different moderating variables.

**Independent variables**	**SMD**	**SE**	**95% CI**	***p***	***I^2^*(%)**	***df***	***Q value and (p) between groups***
**DURATION OF INTERVENTION**							
4–8 weeks	−0.64	0.38	−1.38 to 0.10	0.088	70.83^*^	2	20.59 (**p**<**0.001**)
12 weeks	−1.41	0.41	−2.20 to−0.61	**0.001**	95.53^**^	12
16 weeks	−0.61	0.31	−1.22 to 0.00	**0.050**	85.47^**^	4
5–6 months	−0.23	0.15	−0.52 to 0.07	0.132	62.40^*^	8
1 year	0.11	0.10	−0.08 to 0.30	0.251	0.00	3
**WEEKLY FREQUENCY**							
1	0.11	0.17	−0.21 to 0.44	0.498	20.40	1	13.89 (**0.003**)
2–3	−0.56	0.43	−1.40 to 0.28	0.194	92.62^**^	3
3–4	−0.98	0.26	−1.49 to−0.48	<**0.001**	93.54^**^	20
5	−0.13	0.10	−0.33 to 0.08	0.224	15.97	5
**DURATION OF SINGLE SESSION**							
20–30 min	−0.31	0.19	−0.68 to 0.06	0.105	87.48^**^	14	6.19 (**0.045**)
31–45 min	−1.57	0.49	−2.53 to−0.60	**0.001**	96.72	7
46–65 min	−0.68	0.25	−1.18 to−0.18	**0.007**	83.90^**^	8

*SMD, standardized mean difference; SE, standard error; CI, confidence interval; p, significance value; I^2^, heterogeneity index; df, degrees of freedom. Bold values indicate statistically significant values*.

* *p < 0.05*;

** *p < 0.01*.

## Discussion

The present meta-analysis summarizes evidence on the dose–response relationships between exercise (dose) and improvement of QoL, aerobic capacity, and cardiac function in (response) in older HF patients. The main findings of this meta-analysis were that (i) physical training exerted moderate effects on cardiac function, and small effects on QoL and aerobic capacity in older patients with HF and (ii) the training variable “duration” predicted the effects of physical training on QoL.

### General effectiveness of physical training

Previous meta-analyses have already examined the effect of physical training on QoL and aerobic performance and/or dose–response relationships for training variable (i.e., training intensity) in patients with HF (Pandey et al., 2015; Ostman et al., 2017). Pandey et al. (2015) showed that exercise training improves cardiorespiratory fitness (weighted mean difference = 2.72) and QoL (weighted mean difference = −3.97). These findings are in agreement with the results of Ostman et al. (2017) for QoL. However, as a novelty, our meta-analysis revealed that, aside from small improvements in QoL and aerobic capacity (i.e., 6-MWT), older patients with HF may achieve moderate improvements in cardiac function after physical training.

### Type of training/intervention

Current findings indicate that aerobic training provide moderate improvements in QoL, compared to only small or trivial improvements after combined aerobic and resistance training and resistance training only, respectively. Ostman et al. (2017) reported that combined aerobic and resistance training or aerobic training alone improved QoL, whilst resistance training alone did not improve QoL. Similarly, Mandic et al. (2009) compared aerobic training vs. combined aerobic and resistance training in HF patients and reported that QoL was improved following aerobic training in compliant patients only. Cardiac rehabilitation programs focusing on aerobic training have resulted in reduced symptoms (i.e., pain, lower extremity edema, coughing, and breathe problems) and enhance functional capacity (Pollentier et al., 2010) following implementation.

Concerning changes of aerobic capacity (i.e., 6-MWT), the present meta-analysis revealed that resistance training produced greater performance improvements than aerobic and combined training. This may be explained by the beneficial effects of resistance training on running economy and strength, which results in greater recruitment of type I fibers for the same submaximal load, better muscular coordination, and therefore better mechanical efficiency, whilst likely concomitantly enhancing aerobic capacity (Hartman et al., 2007; Yamamoto et al., 2008, 2010; Cadore et al., 2011). Contrastingly, Wood et al. (2001) reported greater improvements in repeated chair stand after combined training, vs. with resistance-only and endurance-only training in healthy older individuals. Furthermore, another review revealed that resistance and aerobic training have a similar effect on aerobic capacity in older adults (Liu and Latham, 2009). As such, practitioners/physicians should implement resistance training to improve 6-MWT performance, and thus functional capacity in patients with HF.

This meta-analysis showed that aerobic training had the largest effect on cardiac function and mechanisms may be related to presence of mediators such as nitric oxide, which increase cardiac vagal tone after aerobic exercise, and angiotensin II, which inhibits cardiac vagal activity, and the adaptation of the autonomic nervous system in favor of parasympathetic dominance (Kingwell, 2002; Billman and Kukielka, 2007). Previous meta-analysis has suggested that aerobic or combined exercise, but not resistance exercise, may be effective in improving cardiac function in HF patients (Haykowsky et al., 2007). Delagardelle et al. (2002) found a greater improvement of aerobic performance and systolic function after four months of combined aerobic and resistance training than aerobic training in HF patients. Of note, in the present meta-analysis, the absence of significant difference in cardiac function between aerobic and combined training may be due to the high heterogeneity among included studies.

### Effects and dose–response relationships following physical training

#### Training variables (training duration in weeks, weekly training frequency, session duration)

The current meta-analysis substantially advances the literature compared to previous reviews (Piepoli et al., 2004; Smart and Marwick, 2004; Giuliano et al., 2017; Ostman et al., 2017), as we provide the dose–response relationships of physical training variables such as frequency, duration, and volume with training adaptations. Included studies showed large variation in training variables whereby training periods ranged from 4-weeks to 1-year, frequencies from one to five times/week, and duration of training sessions from 10 to 65 min. The characterization of dose–response relationships revealed that, when considered individually, and not as complete training protocol, training periods of 12 weeks, a frequency of 3–4 sessions per week, and durations of 31–45 min of a single training session, were the most effective training variable specifics for improvements in QoL.

Dose-response analyses revealed that shorter training durations are more effective at improving QoL. Specifically, sub-group analysis showed that physical training lasting 12 weeks is most effective for improving global QoL in older HF patients. As illustrated in Table 3, longer training periods produced lower ES, compared to shorter training (i.e., 4–16 weeks) periods. Davidson et al. (2010) reported significant differences between intervention and control groups in the measure of QoL (MLWHFQ) at 3 months, whilst there were no differences between groups at 12 months. Health Canada (1999), American College of Sports Medicine (ACSM) and the American Heart Association (AHA) (Nelson et al., 2007) recommended 30–60 min a day of aerobic activity of moderate-intensity for several months to promote and maintain health, and reduce the risk of chronic disease, premature mortality, functional limitations, and disability. Shorter periods of training are usually accompanied by greater adherence rates (Conraads et al., 2012; De Maeyer et al., 2013). In fact, only minor group could reach the long-term period of >6-month and could reach the 120 min per week (De Maeyer et al., 2013). Despite that, other authors relate improvements of maximal oxygen uptake to the period of training (Tabet et al., 2008) and absence of changes in this parameter is strongly correlated with cardiac risk. With regard to period of training, Lloyd-Williams et al. (2002) found that short-term physical activity is beneficial to patients with chronic heart failure and most patients experience improvement in their QoL.

Our meta-analysis has several limitations that warrant discussion. Firstly, we computed meta-regression and univariate analyses to identify effective dose-response relationships. Of note, findings from univariate analyses must be interpreted with caution because training variables were computed as single factors irrespective of potential between-variable interactions (Gebel et al., 2018). Secondly, we have not performed univariate analyses for aerobic capacity and cardiac function due to the small number of studies. Thirdly, due to limited number of studies examining the effects of physical training in female and male patients, we were not able to compare sexes.

## Conclusions

Physical activity is an effective therapeutic method in older patients with HF, with small to moderate effects on QoL, aerobic capacity, and cardiac function, irrespective of sex and training mode. Dose–response analyses showed that none of the training variables predicted changes in aerobic capacity or cardiac function. However, for QoL, the meta-regression indicated that the training duration significantly predicted the observed improvement, with shorter training duration showing larger improvements.

## Author contributions

MS, RR-C, AP, LH, NB, and MS study concept and design. MS, RR-C, AP, LH, NB, and MS analysis and interpretation of data. MS, RR-C, AP, LH, NB, and MS final approval of the version to be published. MS, RR-C, AP, LH, NB, and MS agreement to be accountable for all aspects of the work.

### Conflict of interest statement

The authors declare that the research was conducted in the absence of any commercial or financial relationships that could be construed as a potential conflict of interest.
